# Notes and Notices

**Published:** 1887-04

**Authors:** 


					﻿NOTES AND NOTICES.
Dr. M. D. Youngman, of Atlantic City, N. J., has associated with him in
partnership Dr. A. W. Baily. While the firm will continue to give careful
.attention to all classes of diseases, it is Dr. Youngman’s intention to devote
himself especially to nervous diseases, both organic and functional.
The Texas Homceopathic Medical Association.—The fourth annual
session will fee held at Fort Worth, Texas, May 3d and 4th, 1887. Officers
for 1887 : President, W. F. Thatcher, M. D., Paris; First Vice-President,
J. R. Pollock, M. D., Fort Worth; Second Vice-President, F. Hines, M. D.,
Corsicana j Secretary pro tern., C. E. Fisher, M. D., Austin ; Treasurer, T. H.
Bragg, M. D., Austin. Legislative Committee: W. F. Thatcher, M. D., ex-
officio ; C. E. Fisher, M. D., M. Slocum, M. D., T. H. Bragg, M. D.; J. Jones,
M.D.
' Danger of Syncope in Hot Baths.—This peril is very great by reason
of-the “determination of blood to the surface of the body, thus quickly
depriving the heart of its normal supply and stimulus.” In cases of mus-
cular weakness of the heart this danger must be imminent whenever the hot
or even the warm bath is used. Apart from this obvious risk, however, there is
always the possibility that in weakly or too impressionable states of the ner-
vous system the peripheral stimulation produced by the application of heat to
the whole of the cutaneous extremities of the afferent nerves may so act upon
the centres as to arrest the evolution of energy by an inhibitory influence.—
New York Medical Times.
				

